# Supplementary material for: Causal Relationship between Aging and Anorexia Nervosa: A White-Matter-Microstructure-Mediated Mendelian Randomization Analysis
Source: Biomedicines. 2024 Aug 16;12(8):1874. doi: 10.3390/biomedicines12081874 (PMC11351342; doi:10.3390/biomedicines12081874)
Supplement: Supplementary file 1 [file biomedicines-12-01874-s001.zip › Supplementary Figure/Figure S8/Leaveoneout plot of MR analysis for TL to ubm-b-1715.pdf]

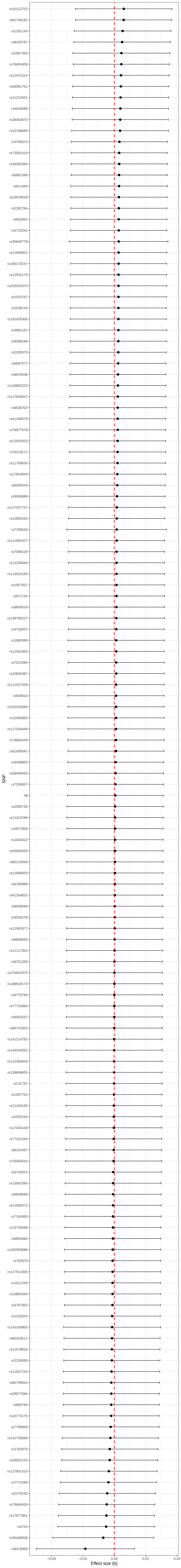
rs10112752
rs61748181
rs1291143
rs8105767
rs2967355
rs78491606
rs12412214
rs56061761
rs11212631
rs4616688
rs28363070
rs10768683
rs3785074
rs73581419
rs34550383
rs6881568
rs611646
rs16978028
rs2282764
rs932002
rs4731541
rs35640778
rs11584821
rs185174247
rs12932179
rs202034370
rs1023767
rs2538745
rs142426306
rs3891167
rs9398196
rs2293579
rs6587577
rs9878436
rs116863223
rs117630647
rs6536702
rs41269079
rs79977579
rs12925933
rs76219171
rs11769630
rs17803849
rs6580343
rs3093888
rs117407747
rs10905255
rs7705526
rs111950327
rs7099229
rs13230646
rs113525195
rs1957937
rs871134
rs9600019
rs139795227
rs4743037
rs1985369
rs12451892
rs7221585
rs10645387
rs111527438
rs939916
rs150150565
rs12369950
rs117034449
rs76666449
rs61405042
rs4498805
rs59409453
rs7209057
All
rs2056726
rs11412296
rs2977608
rs1003322
rs45604339
rs80116508
rs11699829
rs6790988
rs41304832
rs9940099
rs4530278
rs11991877
rs6669563
rs11117354
rs6751209
rs376641875
rs188918174
rs6776756
rs77732866
rs6054257
rs66731853
rs141214782
rs144204502
rs112394493
rs139669835
rs131797
rs1907702
rs11426156
rs2555104
rs17445108
rs77231040
rs8102497
rs76065543
rs5742915
rs13062095
rs6659669
rs11085072
rs7164950
rs73730598
rs9955360
rs182059586
rs762679
rs117512405
rs1611236
rs10805346
rs3767952
rs1332941
rs143190905
rs80324517
rs11579626
rs2230590
rs11557154
rs56799554
rs28577594
rs869785
rs10773176
rs7790856
rs142730696
rs2763979
rs28502153
rs137901416
rs7772289
rs2276182
rs75664430
rs17677991
rs4724
rs35446936
rs9419558
SNP
-0.04
-0.02
0.00
0.02
0.04
Effect size (b)
